# The antimicrobial effects of selenium nanoparticle-enriched probiotics and their fermented broth against *Candida albicans*

**DOI:** 10.1186/2008-2231-22-48

**Published:** 2014-06-06

**Authors:** Erfan Kheradmand, Fatemeh Rafii, Mohammad Hossien Yazdi, Abas Akhavan Sepahi, Ahmad Reza Shahverdi, Mohammad Reza Oveisi

**Affiliations:** 1 Department of Pharmaceutical Biotechnology and Biotechnology Research Center, Faculty of Pharmacy, Tehran University of Medical Sciences, Tehran, Iran; 2 Science and Research Branch, Azad University, Tehran, Iran; 3 Division of Microbiology, National Center for Toxicological Research, U.S. FDA, Jefferson, AR 72079, USA; 4 Department of Medical Biotechnology, School of Advanced Medical Technologies, Tehran University of Medical Sciences, Tehran, Iran; 5 Department of Food and Drug Control, Faculty of Pharmacy, Tehran University of Medical Sciences, Tehran, Iran

**Keywords:** *Candida albicans*, Secretory products, Selenium nanoparticles, Antimicrobial effect, *Lactobacillus plantarum*, *Lactobacillus johnsonii*

## Abstract

**Background:**

Lactic acid bacteria are considered important probiotics for prevention of some infections. The aim of this work was to investigate the effect of selenium dioxide on the antifungal activity of *Lactobacillus plantarum* and *L. johnsonii* against *Candida albicans*.

**Methods:**

*Lactobacillus plantarum* and *L. johnsonii* cells, grown in the presence and absence of selenium dioxide, and their cell-free spent culture media were tested for antifungal activity against *C. albicans* ATCC 14053 by a hole-plate diffusion method and a time-kill assay.

**Results:**

Both *L. plantarum* and *L. johnsonii* reduced selenium dioxide to cell-associated elemental selenium nanoparticles. The cell-free spent culture media, from both *Lactobacillus* species that had been grown with selenium dioxide for 48 h, showed enhanced antifungal activity against *C. albicans.* Enhanced antifungal activity of cell biomass against *C. albicans* was also observed in cultures grown with selenium dioxide.

**Conclusions:**

Selenium dioxide-treated *Lactobacillus* spp. or their cell-free spent broth inhibited the growth of *C. albicans* and should be investigated for possible use in anti-*Candida* probiotic formulations in future.

## Introduction

*Candida albicans,* although it is a commensal yeast in the oral cavity, gastrointestinal tract and urogenital tract, can cause a variety of mild to serious infections. *C. albicans* usually infects immunocompromised patients or others who use antibiotics for a long time [1]. One reason for the overgrowth of *C. albicans* and infection is disequilibrium in the microbiota [2,3]. Probiotics are microorganisms which, when consumed in adequate amounts, can improve intestinal microbial balance and provide benefits for human health [4]. Lactic acid bacteria (LAB) are important probiotics and also part of the normal Gram-positive microflora inhabiting the intestinal mucosa. They aid in prevention of colonization by pathogenic microorganisms [5]. In the vagina, normal *Lactobacillus* species have a critical role in protection against vaginal infections and the transmission of pathogens responsible for sexually transmitted diseases [4-8]. These bacteria produce lactic acid, acetic acid, hydrogen peroxide, and other antimicrobial substances, which allow them to prevent the colonization of pathogens [8,9]. Some LAB strains can protect the human vagina from candidiasis through the production of these exometabolites [10-14]. As yet uncharacterized metabolites from selenium-enriched probiotics have recently been shown to exert an antibacterial effect against *Escherichia coli*[15]. In the present work, we aimed to study the antimicrobial effect of two selenium-enriched *Lactobacillus spp.* cultures and their exometabolites against *C. albicans* ATCC 14053 and to compare these results with anti-*Candida* effects of spent broth of non-selenium-enriched *Lactobacillus* cultures*.*

## Materials and methods

### Microbial strains

*L. plantarum* (ATCC 8014) and *C. albicans* (ATCC 14053) were obtained from the American Type Culture Collection (ATCC). The other *Lactobacillus* was a clinical isolate, which was identified as *L. johnsonii* during a previous study [16].

### Effect of *Lactobacillus* species on selenium dioxide

One hundred milliliters of DeMan–Rogosa–Sharpe (MRS) broth (Merck, Darmstadt, Germany) was used for inoculation of *L. plantarum* and *L. johnsonii* strains*.* The cultures were grown at 37°C in a shaker incubator for 24 h. After this time, selenium dioxide (Merck Schuchardt, Hohenbrunn, Germany) was dissolved in distilled water (289.5 mg/l) and sterilized by a Millipore filter apparatus (Millipore Corporation, Milford, MA, USA). This selenium dioxide solution was added aseptically to each of the *Lactobacillus* cultures to obtain a final concentration of 200 mg/l of Se. The cultures were further incubated at 37°C for 96 h. Two-milliliter samples were withdrawn at zero time and at intervals (24, 48, and 96 h) under aseptic conditions. The bacterial cells were removed from the cultures by centrifugation at 5,000 × *g* for 10 min (Hettich Mikro 200, Tuttlingen, Germany). The supernatant at each time interval was used to measure the concentration of Se remaining in the medium by Somer and Kutay’s spectrophotometric method [17]. The cell pellet from a culture of *L. plantarum* and isolated selenium nanoparticles (SeNPs) were examined at 100 kV by a Philips EM-208 transmission electron microscope (TEM) (FEI Ltd., Eindhoven, The Netherlands) to evaluate the presence and the size of Se NPs deposited inside the *L. plantarum* cells as previously described [18]. To determine the elemental composition of the nanoparticles (NPs), energy dispersive X-ray spectrum (EDX) microanalysis (Vega Tescan, Brno, Czech Republic) was also performed.

### Preparation of *Lactobacillus* cultures for antifungal activity assays

Four flasks, each containing 100 ml MRS broth, were used for inoculation of two sets each of *L. plantarum* and *L. johnsonii* strains*.* The cultures were aerobically grown at 37°C in a shaker incubator for 24 h and one set of each bacterium was treated with selenium dioxide as previously described [16]. The cultures were incubated at 37°C for another 96 h. At zero time and every 24 h, 1 ml samples from all four sets of cultures were harvested under aseptic conditions. The samples were centrifuged at 5000 × *g* for 15 min. All collected supernatants were assayed for antifungal activity against *C. albicans*. Cultures incubated for additional time (96 h) were subjected to further centrifugations to isolate enough culture supernatants for the time-kill assay.

#### *Assay for antifungal activity of L. plantarum and L. johnsonii grown with or without selenium dioxide*

Both a conventional hole-plate diffusion method and a time-kill assay were used to detect antimicrobial activity in the samples. The supernatants of *L. plantarum* and *L. johnsonii,* grown with or without selenium dioxide, were sterilized by filtration through a 0.22 μm Millipore filter. Sabouraud dextrose agar (SDA) plates were inoculated with *C. albicans* and used to test anti-*Candida* effects of the collected supernatants from each culture. 14-mm diameter holes were punched aseptically in each plate and filled with 100 μl of the cell-free supernatants. As a negative control, sterile MRS liquid medium (100 μl) was used. The plates were incubated aerobically for 18 h at 37°C and the diameters of the inhibition zones (mm) were measured.

The effect of cell-free supernatants of 72-h cultures of *L. plantarum* and *L. johnsonii*, grown with or without selenium dioxide, on the survival of *C. albicans* was also evaluated by conventional time-kill assays. To the tubes containing each of the supernatants, *C. albicans* (3 × 10^6^/ml) was added and incubated at 37°C. The viability of *C. albicans* was studied by plating samples taken at different intervals (0.5, 4, 8, 12, and 24 h) on SDA medium and counting the CFU of surviving *C. albicans*.

The antifungal activity of bacterial cells of *L. plantarum* and *L. johnsonii,* grown with or without selenium dioxide, was also assayed with *C. albicans.* Each of the bacterial pellets prepared as previously described was suspended in a normal saline solution containing 3 × 10^6^ CFU/ml of *C. albicans.* The yeast: bacterial ratio in the suspension was approximately 1/1000 CFU/ml. Samples were withdrawn at different intervals (0.5, 4, 8, 12, and 24 h) for determining the number of surviving *C. albicans* in each challenge test. The samples were plated on SDA medium and incubated at 37°C overnight. The surviving *C. albicans* CFU were counted. These experiments were repeated three times.

## Results

### Selenium dioxide reduction

**Figure 1:**
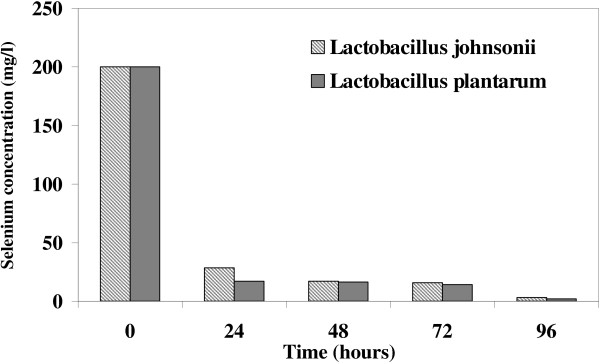
**Reduction of selenium dioxide concentration in cultures of *L. johnsonii *** **or *L. plantarum*, as determined by a spectrophotometric method.**

**Figure 2:**
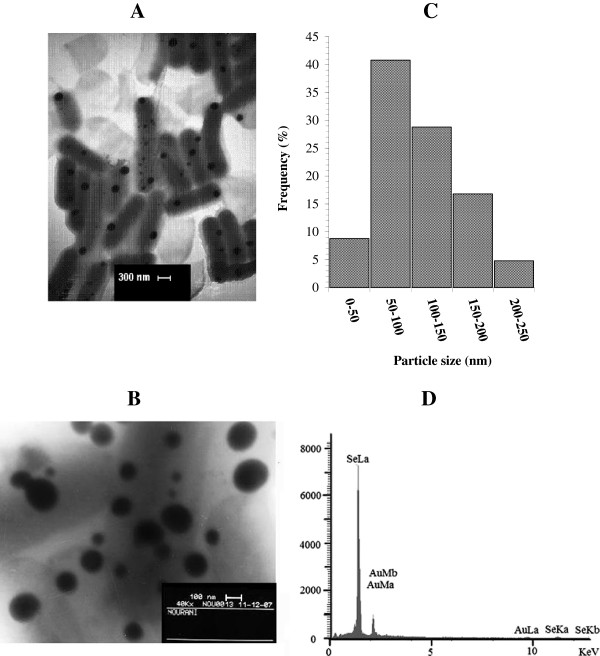
**Characterization of SeNPs-enriched *L. plantarum *** **and purified SeNPs: TEM micrographs (images A and B), Particle size distribution histogram (C) and EDX spectrum of the particles (D).**

After 24 h incubation with both *L. plantarum* and *L. johnsonii,* the concentrations of selenium in the supernatants were considerably reduced. Figure 1 shows the concentration of selenium remaining in the culture medium taken every 24 h. The amount of Se remaining in the supernatants of *Lactobacillus* cultures after 72 h was approximately 3 mg/l, indicating that 98.5% of the selenium ions were reduced in each of the *Lactobacillus* cultures (Figure 1). No appreciable amount of Se was present in the culture supernatant of any of the strains after 96 h of incubation. The selenium-enriched *L. plantarum* cells were selected for a TEM experiment (Figure 2A). Spherical SeNPs of various sizes had formed inside the *L. plantarum* cells (Figure 2B). Figure 2C shows a size histogram of the SeNPs inside the cells; the particle sizes ranged from 25 to 250 nm. Furthermore, EDX microanalysis of the separated NPs exhibited Se absorption peaks consisting of SeLα, SeKα and SeKβ at 1.37, 11.22 and 12.49 keV, respectively (Figure 2D) and confirmed the presence of Se in the samples.

### Anti-*Candida* effect of fermented broth of *L. plantarum* and *L. johnsonii*

**Figure 3:**
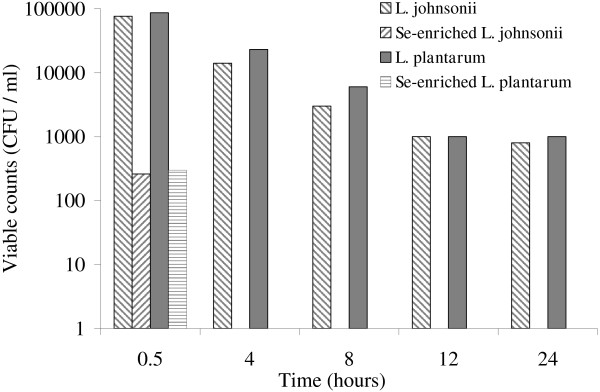
**Viability of *C. albicans *** **after incubation in culture supernatants of *L. johnsonii *** **or *L. plantarum*, grown with or without selenium dioxide.**

The anti-*Candida* effects of the supernatants from *L. plantarum* and *L. johnsonii,* grown with and without selenium dioxide, were compared by measuring the zone of inhibition formed around each of the samples applied to the plates in the hole-plate diffusion method (Table 1). Negligible inhibition zones were observed on the plates containing culture supernatants of either of the species grown without selenium dioxide or sterile MRS broth supplemented with selenium dioxide (200 mg/l). Culture supernatants from *L. plantarum* and *L. johnsonii* grown with selenium dioxide for 48, 72, and 96 h showed potent anti-*Candida* activity and inhibited growth. The maximum antifungal activity was observed in 72 h cultures (Table 1). The time-kill assay, measuring the effect of culture supernatants of each strain, grown with or without selenium dioxide, on the viability of *C. albicans,* confirmed the increase in the antifungal activity of the strains grown with selenium dioxide (Figure 3). Whereas incubation of *C. albicans* with the supernatants of all cultures decreased viability, the number of surviving *C. albicans* cells substantially decreased when incubated with the supernatants of cultures grown for 72 h with selenium dioxide. The difference in the effect, which was observed even after 0.5 h incubation, increased with time. No viable *C. albicans* was present after 4 h incubation with culture supernatants of either species grown with selenium dioxide, but some viable *C. albicans* cells were present even after 24 h incubation with culture supernatants of species grown without selenium dioxide.

**Table 1:** **The antimicrobial activity of spent broth of *L. johnsonii *** **and *L. plantarum *** **cultures grown with or without selenium dioxide against *C. albicans*, as measured by the diameter of the zone of inhibition formed in the hole-plate diffusion assay**

**Supernatant samples**	**Inhibition zone diameter (mm)**			
	**24 h**	**48 h**	**72 h**	**96 h**
*L. plantarum*	-^a^	-	-	-
Selenium-enriched *L. plantarum*	-	23 ± 0.5	28 ± 1	28 ± 0.5
*L. johnsonii*	-	-	-	-
Selenium-enriched *L. johnsonii*	-	22 ± 1	25 ± 0.5	26 ± 0.5
Sterile SeO_2_ supplemented MRS broth	-	-	-	-

^a^Negligible inhibition zone was observed (<9 mm).

### Antifungal effect of *L. plantarum* and *L. johnsonii* cells against *C. albicans*

**Figure 4:**
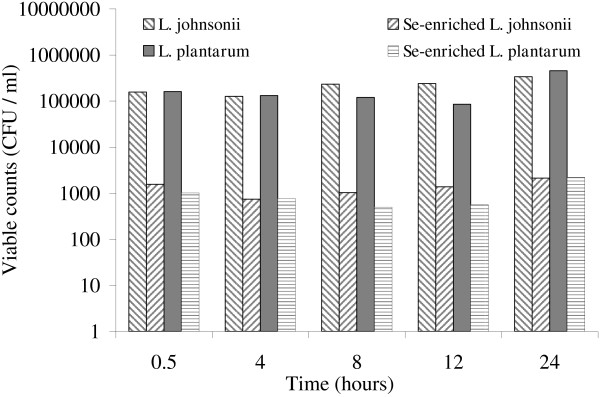
**Viability of *C. albicans *** **after co-incubation with *L. johnsonii *** **and *L. plantarum *** **containing SeNPs.**

The anti-*Candida* effects of the selenium-enriched and non-enriched cells of *L. plantarum* and *L. johnsonii* were also evaluated. The viability of *C. albicans* decreased following co-culture with both bacteria (Figure 4), but cells from selenium-enriched cultures were more effective at killing *C. albicans* (Figure 4). After 0.5 h incubation of *C. albicans* with the *Lactobacillus* strains grown without selenium dioxide, viability of *C. albicans* had decreased by approximately 10-fold, whereas a 1000-fold decrease was seen when incubated with the SeNPs-enriched species (Figure 4). However, the number of viable cells did not decrease with increased incubation time.

## Discussion

Inhibition of *C. albicans* by some strains of *Lactobacillus* species is known and results of clinical trials have shown the effectiveness of some strains of *Lactobacillus* spp. in prevention of *C. albicans* infections [19]. In this study, we have evaluated the interaction of *L. plantarum* and *L. johnsonii* with selenium dioxide on the antifungal activity of these bacteria for *C. albicans.* Both strains converted selenium dioxide to SeNPs of various sizes, which accumulated inside the cells. Whereas both cells and culture supernatants had anti-*C. albicans* activity, substantially higher antifungal activity was observed in the culture supernatants of strains grown with selenium dioxide. It appears that selenium dioxide in the cultures enhanced production of soluble metabolites involved in killing *C. albicans* cells.

The antifungal activity of *L. plantarum* is related to the production of specific compounds, such as phenyllactic acid and 4-hydrophenyllactic acid [20]. When *C. albicans* was mixed with the SeNPs enriched *Lactobacillus* cells or the cell-free fermented broth, clear decreases in viability were observed. The addition of selenium dioxide to the culture medium of either *Lactobacillus* species led to potent increases in antifungal activity against *C. albicans*. Other studies indicate that the adverse effect of LAB can result from production of lactic acid, acetic acid, H_2_O_2_, CO_2_, bacteriocins, and uncharacterized compounds [10-13]. Therefore, selenium dioxide may induce the production of these exometabolites or induce the synthesis of novel anti-*Candida* compounds. At this time, the nature of the exometabolites responsible for the observed antimicrobial activity is not known, and further bioassay-guided fractionation assays should be used to isolate and characterize the active constituent(s).

During the cultivation of *Lactobacillus* spp. in MRS broth containing selenium dioxide, this compound was reduced to elemental SeNPs, which accumulated in intracellular spaces of *Lactobacillus* spp. and may have contributed to the increased antifungal activity of the treated cells.

The modification of the microenvironment of LAB is being considered as a means of preventing infections of the urogenital and intestinal tracts [6-8]. The application of SeNPs enriched- *Lactobacillus* may be a good approach for the design of new strategies in enhancing the activities of probiotics for curing infections caused by urogenital pathogens, such as *C. albicans*.

## Conclusions

The present work assessed the viability of *C. albicans* following exposure to selenium NPs-enriched *Lactobacillus* species or their cell-free culture media. A greater decrease in viability of *C. albicans* was seen for bacteria grown with selenium dioxide than for non-Se-enriched bacteria. A direct antifungal effect was observed when SeNPs-enriched *Lactobacillus* spp. were co-cultured with *C. albicans.* In addition, evidence for release of potent exometabolites was indicated by the strong inhibition of growth of *C. albicans* treated with cell-free culture media from the SeNPs-enriched *Lactobacillus* species. This is the first time in which antifungal activity of the combination of *Lactobacillus* spp. and selenium has been studied. This phenomenon should be further evaluated for its practical application.

## Competing interests

The authors declare that they have no competing interests.

## Authors’ contributions

EK: Conducted experiments. FR: Supervised antifungal experiments and revision of manuscript. MHY and AAS: Participated in microbiological experiments. ARS: Project design and manuscript preparation. MRO: Participated in discussion on experimental procedures. All authors read and approved the final manuscript.
